# From antimicrobial to anticancer: unraveling the potential of pleurocidin and pleurocidin-derived peptides in the treatment of cancers

**DOI:** 10.3389/fphar.2024.1340029

**Published:** 2024-01-26

**Authors:** Ewelina Piktel, Urszula Wnorowska, Joanna Gorbacz-Konończuk, Jakub Sienkiewicz, Katarzyna Głuszek, Sławomir Okła, Robert Bucki

**Affiliations:** ^1^ Independent Laboratory of Nanomedicine, Medical University of Bialystok, Bialystok, Poland; ^2^ Department of Medical Microbiology and Nanobiomedical Engineering, Medical University of Bialystok, Bialystok, Poland; ^3^ Collegium Medicum, Jan Kochanowski University in Kielce, Kielce, Poland

**Keywords:** antimicrobial peptides, pleurocidin, pleurocidin derivatives, anticancer drugs, drug development

## Abstract

Antimicrobial peptides (AMPs), commonly referred to as host defense peptides, are found in a wide range of organisms, including bacteria, plants, and both vertebrate and invertebrate animals. They function as an initial defense mechanism against pathogenic microorganisms, modulate immune responses, and in specific instances, confer protection against the onset of cancer. Pleurocidin (Ple) is a linear antimicrobial peptide with amphipathic α-helical conformation, isolated originally from the winter flounder (*Pleuronectes americanus*), notable for its wide-ranging effectiveness against both bacteria and fungi. While the majority of research on pleurocidin’s biological characteristics has primarily focused on deciphering its mechanisms of interaction with the biological membranes of pathogenic bacteria and host cells, as well as investigating its modes of killing activities, there is a growing body of evidence suggesting that pleurocidin and pleurocidin-derived analogs might be effectively employed as anti-cancer agents against breast carcinoma and leukemia due to their potent cytotoxic properties and selectivity towards cancer cells. Notably, some characteristics of pleurocidin observed in microbiological investigations of this compound could be effectively applied in examining the anti-cancer capabilities of Ple-like derivatives. This review provides a comprehensive overview of the literature on the biological activities of pleurocidin, pleurocidin-derived peptides, pleurocidin-containing hybrid peptides, and nanosystems. The primary emphasis is on elucidating the range of activities exhibited by these compounds, evaluating their potential therapeutic applications, assessing their safety profile, and identifying any limits observed thus far. This paper will also discuss potential areas for further investigation into the anti-cancer effects of Ple and its derivatives, drawing insights from microbiological research.

## 1 Introduction

Antimicrobial peptides (AMPs) are linear and cyclic bioactive compounds with 10–100 amino acid residues that are synthesized by a majority of living organisms, ranging from prokaryotes to mammals, and serve as a crucial element of their innate defense mechanisms (Ageitos et al., 2017). While their main function is to provide host defense by exerting lethal effects on invading pathogenic microbes, a compelling amount of data demonstrates that they also serve as immunological modulators and anti-cancer compounds in higher species (Kang et al., 2019; Kordi et al., 2023). Despite the substantial heterogeneity of antimicrobial peptides in terms of their structural and biological characteristics, certain shared attributes have been discerned. These include their relatively small size, the presence of cationic and hydrophobic regions, amphipathic stereo geometry, and non-specific mechanisms of action (Pushpanathan et al., 2013). Other key characteristics include targeted cytotoxic activity towards the membranes microorganisms compared to eukaryotic cells, reduced toxicity compared to antibiotics, and a lower likelihood of development of resistance. This makes AMPs promising and possible future pharmaceutical contenders (Ageitos et al., 2017; Kordi et al., 2023). Moreover, a sub-class of endogenous host peptides, so-called anticancer peptides (ACPs) have been distinguished and clinically highlighted due to their plethora of unique, anti-tumorigenic characteristics, such as induction of intracellular cell death mechanisms, particularly apoptosis, suppressing the formation of tumor blood vessels or regulation of immune protective mechanisms (Chiangjong et al., 2020; Xie et al., 2020). Certain limitations related to the clinical implementation of some endogenous peptides, such as the expense of synthesis, stability difficulties, the diminished efficacy of certain peptides in specific *in vivo* circumstances, or unsatisfactory selectivity against cancer cells were identified (Greber and Dawgul, 2017; Chiangjong et al., 2020; Wang et al., 2021). Regardless, several of them, mostly synthetic analogs, have undergone successful clinical trials and have received FDA approval as therapeutics against bacterial infections and cancers (Micale et al., 2014; Chen and Lu, 2020; Chiangjong et al., 2020). This substantiates the considerable potential exhibited by this class of compounds. Consequently, ongoing research efforts are dedicated to the discovery of novel physiologically active molecules, with a focus on enhancing their selectivity of action and minimizing potential toxicity (Wang et al., 2021; Karami Fath et al., 2022). This article provides a comprehensive overview of the existing research regarding pleurocidin (Ple), an antimicrobial peptide derived from the tissues of winter flounder, as well as synthetic peptides that have been developed based on the chemical nature of pleurocidin. Although Ple is most recognized as an agent with broad-spectrum antimicrobial activities (Tao et al., 2011; Lee and Lee, 2016; Ko et al., 2019), an ever-growing number of evidence demonstrates the potential of Ple in the treatment of cancer (Ebrahimdoost et al., 2023). Particularly, for pleurocidin-derived peptides, which have been reported to have enhanced biological activity against both bacterial and mammalian cells, clinical applications have been highlighted. Potential directions of research on the anticancer properties of Ple and its derivatives are also discussed and hypothesized based on the extrapolation of research focused on microorganisms.

## 2 Structure and physicochemical features of pleurocidin

**FIGURE 1:**
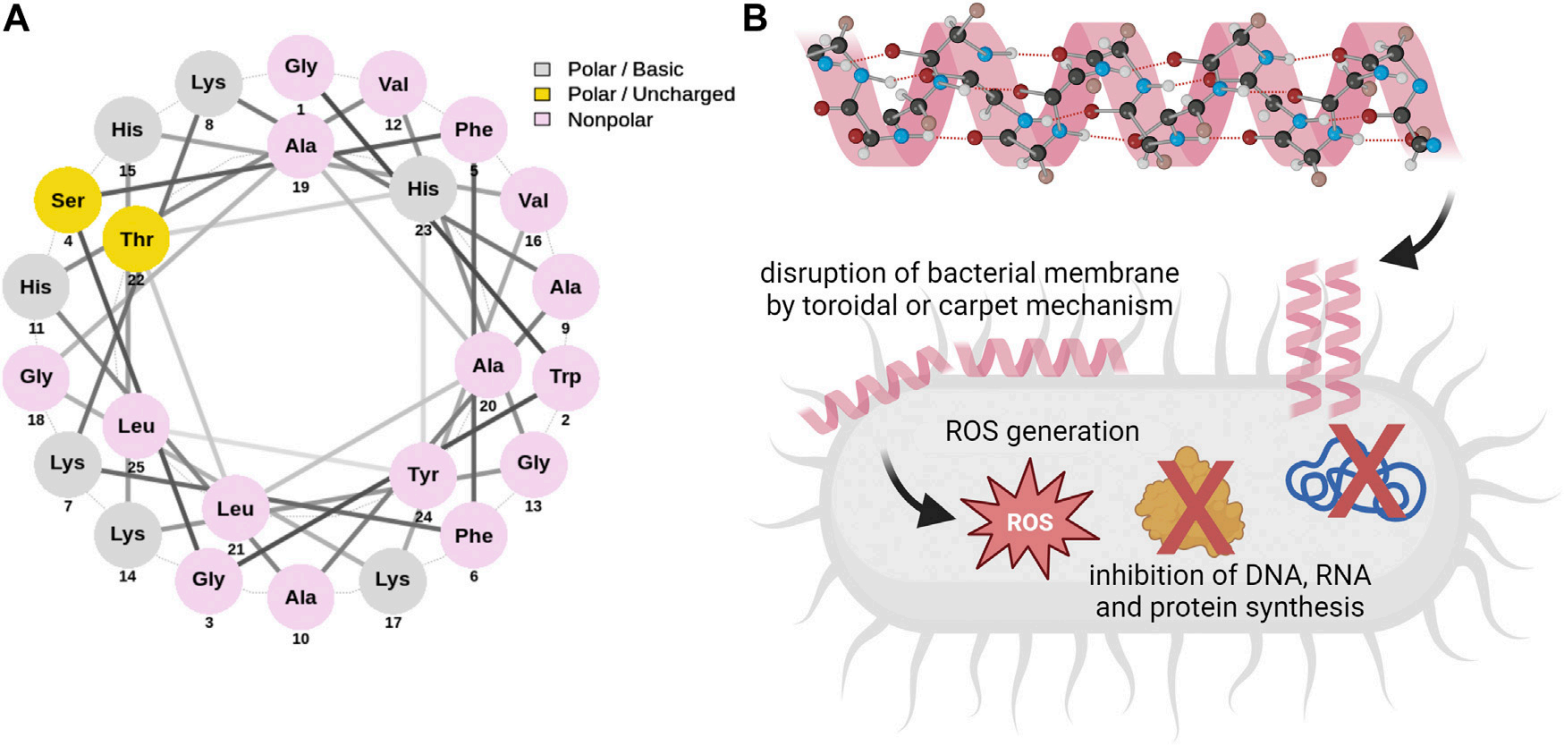
Helical wheel diagram of pleurocidin **(A)** and basic molecular mechanisms determining the antimicrobial activities of Ple **(B)**. The three-letter abbreviations of amino acids were used and their color classifications are as follows: grey (polar basic), yellow (polar uncharged), and pink (nonpolar). Pleurocidin is characterized by α-helical conformation and was proven to interact with bacterial membranes by either toroidal or carpet mechanisms. Dual mechanisms involving membrane perturbation, excessive ROS generation, and inhibition of intracellular molecules’ synthesis were demonstrated to participate in the antimicrobial effects of pleurocidin. The helical wheel diagram was generated using NetWheels online software (Mól et al., 2018). Schematic diagrams on panel B were prepared using Biorender.com.

Pleurocidin is a linear antimicrobial peptide with amphipathic α-helical conformation consisting of 25 amino acid residues (sequence: GWGSFFKKAAHVGKHVGKAALTHYL) isolated for the first time in 1997 by Cole *et al.* from the winter flounder (*Pleuronectes americanus*) (Cole et al., 1997), formed from a 68-residue prepropeptide under proteolytic cleavage (Cole et al., 2000). Based on phylogenetic analyses, pleurocidin was shown to comprise a single cecropin superfamily together with dermaceptin and ceratotoxin families of antimicrobial peptides (Cole et al., 1997; Tamang and Saier, 2006). While the initial data demonstrated that pleurocidin is produced in the epidermal mucous cells, where is stored and subsequently released under appropriate stimulation (Cole et al., 1997), it was later revealed that goblet cells of the flounder small intestine, as well as eosinophilic granular cells of winter flounder gill, are also a source of this peptide (Cole et al., 2000; Murray et al., 2003). Further experiments demonstrated that pleurocidin gene expression might be regulated in response to infection and inflammation (Cole et al., 2000). Pleurocidin was predicted to have an amphiphilic α-helical conformation with hydrophilic and hydrophobic residues on opposing surfaces of the helical structure, comparable to some other antimicrobial peptides (Cole et al., 1997; Syvitski et al., 2005; Lan et al., 2010). The toroidal or carpet mechanism (Yoshida et al., 2001; Saint et al., 2002; Syvitski et al., 2005; Mason et al., 2006; Talandashti et al., 2019; Talandashti et al., 2021) followed by induction of oxidative stress and/or inhibition of intracellular molecules in treated pathogens (Choi and Lee, 2012) were demonstrated as the main mechanisms of pleurocidin activities (Figure 1). Particularly, this non-specific, membrane-targeting mode of action assures the great potential of pleurocidin and pleurocidin-derived peptides as components with a broad spectrum of biological activities, justifying further investigation into the anti-cancer properties of these molecules.

## 3 Antimicrobial activities of pleurocidin. Potential translation of antimicrobial activities of pleurocidin into anticancer ones

To date, pleurocidin and pleurocidin-derived peptides have been acknowledged for their broad-spectrum efficacy in combating bacterial diseases that affect both humans and animals (Table 1). In one of the first studies, pleurocidin was demonstrated to display potent bacteriostatic and bactericidal activities against different fish-, sheep-, and human pathogens with estimated minimal inhibitory concentrations (MICs) ranging from 1.1 to >35 μg/mL [0.4 to >1.9 µM] (Cole et al., 1997). Later, its biocidal activities were confirmed against clinically relevant bacterial and fungal pathogens, including *Pseudomonas aeruginosa* and *Acinetobacter baumannii* (Cole et al., 2000; Ko et al., 2019). Pleurocidin and Ple-derived peptides were also demonstrated to be active against cariogenic bacteria, including *Streptococcus mutans*, *S. sanguinis,* and *S. sobrinus* strains (Tao et al., 2011; Zhang et al., 2016), drug-resistant *Staphylococcus aureus* and Gram-negative pathogenic bacteria (Ko et al., 2019). Several reports have also demonstrated the activity of these peptides against fungal representatives–mostly yeast from the *Candida* genus (Jung et al., 2007; Sung and Lee, 2008), but also against *Saccharomyces cerevisiae*, *Trichosporon beigelii* (Jung et al., 2007; Sung and Lee, 2008), or filamentous phytopathogenic fungi (Souza et al., 2013). To date, Plc-2 peptide, an l2-amino acid fragment from the C-terminus of Plc was identified as the core pleurocidin fragment retaining full antibacterial activity, and thus, the shortest Ple-derived peptide with antimicrobial action (Souza et al., 2013). Moreover, pleurocidin was demonstrated to act synergistically with D-cycloserine against *Mycobacterium smegmatis* (Cole et al., 2000) as well as with bacteriocins from lactic acid bacteria against *Escherichia coli* (Lüders et al., 2003)*.* It provides a clear demonstration of the significant enhancement in specific activity and expanded target-cell range of peptides by the synergistic combination of eukaryotic and prokaryotic antimicrobial peptides (Lüders et al., 2003). Furthermore, pleurocidin is not affected by physiological levels of magnesium and calcium, which have been reported to hinder the activity of the mammalian antimicrobial peptide defensin, possibly by increasing bacterial membrane stabilization (Coughlin et al., 1983) as well as resistant to elevated NaCl concentrations (Douglas et al., 2001). This strongly supports the statement on the broad-spectrum activity of pleurocidin, determined mainly by its membrane-permeabilizing activities. Notably, pleurocidin and its derivatives were recorded to be effective against both antibiotic-susceptible and multidrug-resistant Gram-negative and Gram-positive bacterial isolates (Hsu et al., 2022), indicating the efficiency regardless of drug resistance mechanisms or target membrane composition. In effect, some assumptions were made that Ple and Ple-like peptides would be cytotoxic against a spectrum of cancer cells, regardless of genetic and phenotypic fluctuations between them, and indeed, some already published studies confirm this hypothesis (Hsu et al., 2022).

**TABLE 1:** Antimicrobial activities of pleurocidin and pleurocidin-derived peptides. Denotation of peptides’ abbreviations are explained more detailed in the main text. Full amino acid sequences for all developed peptides are demonstrated in the original reports. * indicate that concentration corresponding to MIC value was converted to µM for the clarity of data presentation.

Peptide	Tested pathogens	MIC range	Proposed mechanism of action	Reference
Pleurocidin	*E. coli, Serratia marcescens, Bacillus subtilis, P. aeruginosa, S. aureus, Salmonella typhimurium I, S. typhimurium II*	0.4 to >1.9 [µM]*	Interaction and disruption of membranes	Cole et al. (1997)
Pleurocidin	*P. aeruginosa, Klebsiella pneumoniae, S. aureus, C. albicans, M. smegmatis*	4.7 to >37 [µM]*	Interaction and disruption of membranes	Cole et al. (2000)
Pleurocidin	*E. coli*	5.9 [µM]*	Interaction and disruption of membranes. Facilitating of access to intracellular targets for other AMPs	Lüders et al. (2003)
Pleurocidin	*S. mutans, S. sanguinis, S. sobrinus*	3–11.8 [µM]*	Interaction and disruption of membranes	Zhang et al. (2016)
Pleurocidin	*S. mutans, S. sanguinis, S. sobrinus, S. gordonii, Lactobacillus acidophillus, L. casei, L. fermenti, Enterococcus faecalis*	0.7 to >94.4 [µM]*	Interaction and disruption of membranes	Tao et al. (2011)
Pleurocidin	*S. aureus, Listeria monocytogenes, B. subtilis, P. aeruginosa, A. baumannii, E. coli*	1–4 [µM]	Interaction and disruption of membranes Binding to DNA and causing interference with cellular functions	Ko et al. (2019)
Ple-amide	*S. aureus, S. xylosus, L. monocytoenes, S. bovis, E. coli, Enterobacter aerogenes, E. cloacae, Yersinia enterocolitica, P. aeruginosa, S. enterica, K. oxytoca, E. faecium, K. pneumoniae, A. baumannii*	0.4–11.8 [µM]*	Interaction and disruption of membranes	Hsu et al. (2022)
Pm1-Pm15 analogues	*S. mutans, S. sanguinis, S. sobrinus*	4.2 to >250 [µM]*^a^	Interaction and disruption of membranes	Zhang et al. (2016)
Pleurocidin Anal-S, Anal-R analogues	*C. albicans, S. cerevisiae, T. beigelii*	2.5–10 [µM]	Interaction and disruption of membranes	Sung and Lee (2008)
L-Ple, D-Ple	*C. albicans, S. cerevisiae, T. beigelii*	2.5–5 [µM]	Interaction and disruption of membranes	Jung et al. (2007)
Pleurocidin, Anal 1–4 peptides	*S. aureus, E. faecius, E. coli* *P. aeruginosa*	2.5–40 [µM]	Interaction and disruption of membranes	Cho et al. (2012)
Plc-2	*P. aeruginosa, E. coli, S. aureus, Alternaria* sp.*, Fusarium oxysporum, Aspergillus niger, A. ochraceus, Cladosporium fulvum, Colletotichum* sp.	2.3 to >37.4 [µM]*	Interaction and disruption of cytoplasmic and nuclear membranes	Souza et al. (2013)
Pleurocidin and Ple-AG/GA/AA analogues	*E.coli, p. aeruginosa, S. typhimurium, B. subtilis, S. epidermidis, S. aureus*	0.5–4 [µM]	Interaction and disruption of membranes	Lim et al. (2004)
GK-2–GK-4 peptides	Drug-susceptible and drug-resistant *S. aureus, E. faecalis, E. coli, S. enteritidis, A. baumannii, P. aeruginosa, P. cibarius*	1.1–16.9 [µM]*^b^	Interaction with bacterial membrane-specific components, membrane depolarization and promotion of ROS generation	Wang et al. (2022)
P-Der	*E. coli*	0.7 [µM]*	Interaction and disruption of membranes. Inhibition of macromolecular synthesis	Patrzykat et al. (2002)
IMB-1–IMB-3	*S. mutans*	2.2–44 [µM]	Interaction and disruption of membranes	Mai et al. (2011)
NRC-01–NRC-20	*A. salmonicida, S. enterica, P. aeruginosa, E.coli*	0.3 - > 58.9 [µM]*^c^	Interaction and disruption of membranes	Patrzykat et al. (2003)

^a^ For calculations, molecular weights of Pm11 and Pm2 were used.

^b^ For calculations, molecular weight of GK-2, was used.

^c^ For calculations, molecular weights of NRC-03, and NRC-02, were used.

Previous studies revealed that pleurocidin exhibits bactericidal properties through two mechanisms: membrane depolarization and rupture, as well as translocation inside bacterial cells, hence exerting antimicrobial actions intracellularly (Patrzykat et al., 2002). This was encouraged by the analyses demonstrating no correlation between peptide membrane translocation and calcein release (Yoshida et al., 2001). In a study by Patrzykat et al., 2002 pleurocidin when present at its minimum inhibitory concentrations, exhibited a reduced propensity for causing damage to cell membranes but retained their capacity to effectively inhibit macromolecular production, i.e., DNA, RNA. It was concluded that the capacity of the peptide to translocate across the membrane and into bacterial cells without evident membrane permeabilization allowed such an effect. This is in accordance with the research by Pellegrini et al., 2000 demonstrating that the presence of short peptides with a positive charge (lysozyme in this case) effectively hindered the processes of DNA and RNA synthesis in *E. coli* before the occurrence of inner membrane permeabilization (Pellegrini et al., 2000). Moreover, in independent studies, pleurocidin was found to possess DNA binding capabilities (Lan et al., 2010; Ko et al., 2019), although this particular characteristic alone does not fully account for the peptide’s significant antibacterial effectiveness (Lan et al., 2010). The GK-4 peptide, developed by truncating 11 amino acid residues at the C-terminal of pleurocidin with subsequent substitution of some of the residues, was demonstrated to interact with bacterial membrane components and then, promote the overproduction of ROS, leading ultimately to cell death (Wang et al., 2022). Likewise, as a mechanism of fungicidal activity of pleurocidin and pleurocidin-like peptides, the overproduction of reactive oxygen species followed by induction of oxidative stress, mitochondrial membrane depolarization, apoptosis induction, and subsequent membrane disruption was indicated (Cho and Lee, 2011). In another study, bactericidal effects of Ple were concluded to result from the induction of caspase-like and RecA-mediated induction of intracellular apoptosis-like death (ALD) (Lee and Lee, 2016). Ho et al., 2016 revealed also the effect of an amidated hybrid of the flounder pleurocidin and the frog dermaseptin (P-Der) on the inhibition of the bacterial catabolic processes. In a separate investigation, the utilization of molecular modeling and docking analysis data revealed the ability of pleurocidin-like peptides to selectively target enterotoxin H derived from *Klebsiella pneumoniae*, which suggests the potential of these peptides to interact with non-membrane components (Bupesh et al., 2019). Collectively, these reports provide strong support for the assertions regarding the impact of pleurocidin and its effects on diverse intracellular processes, including the induction of apoptosis.

At the same time, strong evidence from molecular basis tests has proven some membrane-selectivity of pleurocidin. It is highly favorable when fighting infections as the safety of applied antimicrobial therapy increases, but at the same time, such a phenomenon might contribute to the enhanced killing of cancer cells (Yoshida et al., 2001; Talandashti et al., 2019). For instance, Yoshida et al., 2001 demonstrated that pleurocidin had a low affinity for neutral phospholipid bilayers while demonstrating a high affinity for acidic phospholipids. In another study, the utilization of all-atom molecular dynamics (MD) simulations also demonstrated that the peptide exhibited a reduced level of interaction with neutral phospholipid bilayers composed of DOPC (serving as an artificial zwitterionic membrane model) and subsequently experienced a loss of its secondary structure. In contrast, an increased level of Ple engagement resulting in preserving its α-helical conformation of Ple was demonstrated for phospholipids that carry a negative charge which is due to electrostatic interaction with Lys amino residues of pleurocidin (Talandashti et al., 2019). This is consistent with the results of the other analysis indicating that Pleurocidin had a greater impact on the arrangement of anionic phospholipids compared to zwitterionic phospholipids (Mason et al., 2006; Talandashti et al., 2019). Ple in several studies was also demonstrated as displaying low hemolytic activities against human erythrocytes, which can be attributed to the high abundance of cholesterol within the membrane of red blood cells (Ko et al., 2019; Talandashti et al., 2019). Such orchestrated membrane-selectivity of pleurocidin would be advantageous in enhancing the safety of tumor-targeting therapeutic approaches. Compelling evidence shows that cancer cells are characterized by negative charges on surface cells, which are dynamically regulated by glycolytic capacity (Riedl et al., 2011; Deng et al., 2022) and presentation of a spectrum of negatively charged molecules (N'Guessan et al., 2020), while normal cells are either charge-neutral or slightly positive. Accordingly, when comparing the cytotoxicity of Ple and Ple-like peptides against cancerous and non-cancerous cells, it has been indicated that IC_50_ values against malignant cells (i.e., the concentration required to inhibit the activity of 50% of the cell population) can be up to several times lower than those required to achieve toxic effects in normal cells (Hsu et al., 2022).

## 4 Optimization of pleurocidin molecules to improve their biological functions

Presently, considerable efforts are undertaken to enhance the biological efficacy and alleviate toxicity characteristics of pleurocidin, with the ultimate objective of attaining optimal performance (Table 2). To date, several native peptide modifications, including substitution of certain amino acids (Sung and Lee, 2008), removal of residues that have not been proven to be significant for biological activity (Zhang et al., 2016), enantiomers synthesis (Jung et al., 2007), or extension of the peptide with additional amino acids or functional groups (Bryksa et al., 2006; Hsu et al., 2022) have been demonstrated to obtain a series of peptide derivatives with antimicrobial, antifungal and anti-cancer activities. Unique derivatives are also fusion peptides developed from two individual fragments of separate peptides. In this particular context, Mai et al., 2011 formulated peptides that incorporated the targeting domain of *S. mutans* ComC signaling peptide (CSP) and different domains of NRC-4 (a pleurocidin variation as elaborated in subsequent sections) to produce fusion peptides that specifically target *S. mutans* strains. In another study, a hybrid of pleurocidin and dermaseptin (indicated as P-Der) was synthesized and tested as an antimicrobial against *E. coli* (Ho et al., 2016).

**TABLE 2:** Modifications of pleurocidin to improve antimicrobial and anti-cancer activities of Ple. Amino acid sequence of native pleurocidin is presented in the first row of the table. Bold letters in amino acid sequences of Ple-derived peptides indicate alterations when compared to the native peptide composition. Residues shown in italic are D-amino acids. For the clarity of the Table, amino acid sequences of the most active derivatives are demonstrated.

Peptide(s)	Amino acid sequence(s)	Modification of Ple	Effect of modification	Biological activity of peptide	Ref.
Pleurocidin	GWGSFFKKAAHVGKHVGKAALTHYL				
Pm1–Pm15	W**FK**FFKK**FFKKW**K (Pm11)	Replacement of some amino acids from Ple (1–18) C-terminal region with lysine or phenylalanine	Development of 15 analogue peptides with 10–19 amino acids in length	Variable activity against cariogenic bacteria from *Streptococcus* genus	Zhang et al. (2016)
		Removal of amino acids that are not directly involved in antibacterial activity	Modification of hydrophobicity, hydrophobic moment and net charge		
Anal-R and Anal-S	**R**W**R**SFFKKAAH**R**GKHVGK**R**A**R**THYL (Anal-R) **S**W**S**SFFKKAAH**S**GKHVGK**S**A**S**THYL (Anal-S)	Arg (R) or Ser (S)-substitution at the hydrophobic face of Ple	For Anal-S and Anal-R: Decreased net hydrophobicity	Potent antifungal activity with decreased hemolysis	Sung and Lee (2008)
			For Anal-R: Increased cationicity		
			Decreased the α-helical conformation		
D-Ple	*GWGSFFKKAAHVGKHVGKAALTHYL*	Substitution of L-amino acids to D-amino acids in the whole Ple sequence	Enantiomer	Resistance to proteases	Jung et al. (2007)
r-pleurocidin-G	GWGSFFKKAAHVGKHVGKAALTHYL**G**	Extension the pleurocidin sequence with a C-terminal glycine	Stabilization of α-helix	Intensification of antibacterial activity	Bryksa et al. (2006)
Plc-2	KHVGKAALTHYL	Removal of certain amino acids from N-terminal Ple sequence	Obtaining the shortest Ple fragment with cytolytic activity	Antibacterial and antifungal	Souza et al. (2013)
Ple-amide	GWGSFFKKAAHVGKHVGKAALTHYL-**NH2**	Amidation of C-terminal fragment	Increase of molecular charge	Increased antimicrobial and anticancer activity	Hsu et al. (2022)
Ple-AG/GA/AA	GWGSFFKKAAHV**A**KHV**A**KAALTHYL (Ple-AA)	Substitution of glycines in 13 and 17 position of Ple at alanine	Increase in α-helical content	Increased hemolysis	Lim et al. (2004)
GK-2–GK-4	GW**KK**FFKK**WK**HV**W**K (GK-4)	Truncation 11 amino acids at the C-terminal Replacing glycine with tryptophan and lysine	Improvement of α-helical and amphiphilic properties	Resistance to proteases	Wang et al. (2022)
			Reduced synthesis costs	Antibacterial activity	
IMB-1–IMB-3	**TFFRLFNR-GG-**GWGSFFKKAAHVGKL-NH2 (IMB-2)	Fusion of targeting domain of CSP and portions of NRC-04	Target specificity	Salt resistance	Mai et al. (2011)
				Activity against *S. mutans*	
NRC-03D	G*RRKRK*WL*RRI*G*K*GV*K*IIGGAALDHL-NH2	Substitution to D-lysine and D-arginine	Enhanced stability	Decreased activity against leukemia cells	Morash et al. (2011)
[D]-NRC-03	*GRRKRKWLRRIGKGVKIIGGAALDHL*-NH2	Substitution of all residues to D-amino acids	Enhanced stability	Resistance to proteases	Hilchie et al. (2015)
				Increased activity against breast cancer cells	
p28-NRC	n/a	Linking p28 and NRC proteins	Obtaining of chimeric protein	Increased activity against breast cancer cells	Soleimani et al. (2019)

The existing body of literature provides valuable information regarding the role of individual amino acids in a particular region of Ple. Nevertheless, certain contradictory effects on the biological activities of developed derivatives were reported. Cho et al., 2012 stated that both the N- and C-terminal regions of pleurocidin are critical for its antibacterial activity as truncated peptides obtained by partial deletions in those fragments were characterized by reduced biocidal activities due to a decrease in net hydrophobicity. Regardless, Souza et al., 2013 demonstrated that the core pleurocidin fragment retaining full antibacterial activity was located on the C-terminus. In contrast, when related to ROS-mediated fungicidal activity, the hydrophobic amino acids in the N-terminal region of Ple are more crucial for antifungal activity than those in the C-terminal region (Lee and Lee, 2010; Choi and Lee, 2013). Those contradictory studies might hamper the understanding of the role of certain sequences in the biological activities of peptides, nevertheless, some tendencies are seen when comparing amino acid sequences of developed derivatives. Accordingly, a majority of most active peptides is truncated by the C-terminus, highlighting the significance of amino acid residues in the N-terminal of Ple for peptide structure and activity (Lim et al., 2004; Sung and Lee, 2008; Zhang et al., 2016; Wang et al., 2022). Accordingly, *in silico* analyses demonstrated also that the motifs (A15_B) of amino acid positions 2–19 in pleurocidin are those on which the focus should be centered due to their high stability and potential highest antimicrobial activity (Okella et al., 2020). When considering the potency of insertion of the peptides into the membranes, Trp-2, Phe-5, and Phe-6 residues, located in the N-terminal region of the Ple were also highlighted (Talandashti et al., 2019). Indeed, a comparative analysis of amino acid sequences revealed that residues in these positions (as well as Phe-7) are constant in all derivatives.

In another study, Zhang et al., 2016 prepared a series of pleurocidin-derived peptides based on the Ple (1–18) region as an original template and replaced several amino acids with lysine and phenylalanine to obtain a satisfactory activity against *Streptococcus* cariogenic bacteria. The antimicrobial activities of derivatives were found to be primarily influenced by hydrophobicity rather than overall charge. Nevertheless, as supported by the evidence, simply increasing the hydrophobicity may not be enough when the peptide is too short and consequently, may not be able to fully insert into the bilayer and form significantly large pore channels (Zhang et al., 2016). This finding suggests that the act of removing certain amino acids to shorten a peptide may not always yield beneficial outcomes, despite the potential advantages it offers in terms of reducing synthesis costs. Accordingly, when comparing the amino acid sequences of developed derivatives, a majority of them range in length from 13 to 18. To date, a Plc-2 peptide, being an l2-amino acid, the C-terminal fragment of pleurocidin was evidenced as the shortest one with similar properties to its parent compound (Souza et al., 2013).

It was also revealed that α-helicity had a greater impact on the actions of the peptide compared to hydrophobicity (Yoshida et al., 2001). The alteration of this parameter may yield advantageous outcomes for innovative therapeutics, although it is important to exercise caution in this regard. The enhanced conformational flexibility of pleurocidin leads to a significantly modified structure, allowing for greater membrane penetration at lower peptide-to-lipid ratios, and thus more potent bactericidal effects (Amos et al., 2016). Nevertheless, it is important to note that this alteration may also lead to an increase in hemolysis compared to the native peptide (Lim et al., 2004). The biological activity of pleurocidin is also considerably altered upon D-amino acids substitution to obtain proteolytic resistance. As evidenced, the effect of this modification depends on the membrane composition of target cells (Lee and Lee, 2008; Lee et al., 2009), since D-enantiomers were less active against bacterial pathogens, but exerted increased fungicidal effects (Lee et al., 2009). Data on the anti-cancer effects of Ple-derived peptides synthesized by the replacement of some or all residues to D-amino acids are also contradictory (Hilchie et al., 2011; Morash et al., 2011). This also implies that in the process of synthesizing derivatives for use in a particular biological context, it is crucial to conduct comprehensive investigations into the characteristics of the original peptide and the impact of performed modifications on the outcome. This is because the specific biological membrane of the target cell or intracellular factors may necessitate the consideration of alternative physicochemical parameters that are most pertinent.

## 5 Anti-cancer potential of pleurocidin and pleurocidin-derived peptides

The non-specific, membrane-based mechanism of the biocidal features of pleurocidin encouraged the hypotheses on the cytotoxic activities of this group of compounds. Thus far, there has been substantial evidence presented regarding the extensive range of anti-cancer activities exhibited by pleurocidin and pleurocidin-derived peptides (Hilchie et al., 2011; Hilchie et al., 2013; Hilchie et al., 2020).

**FIGURE 2:**
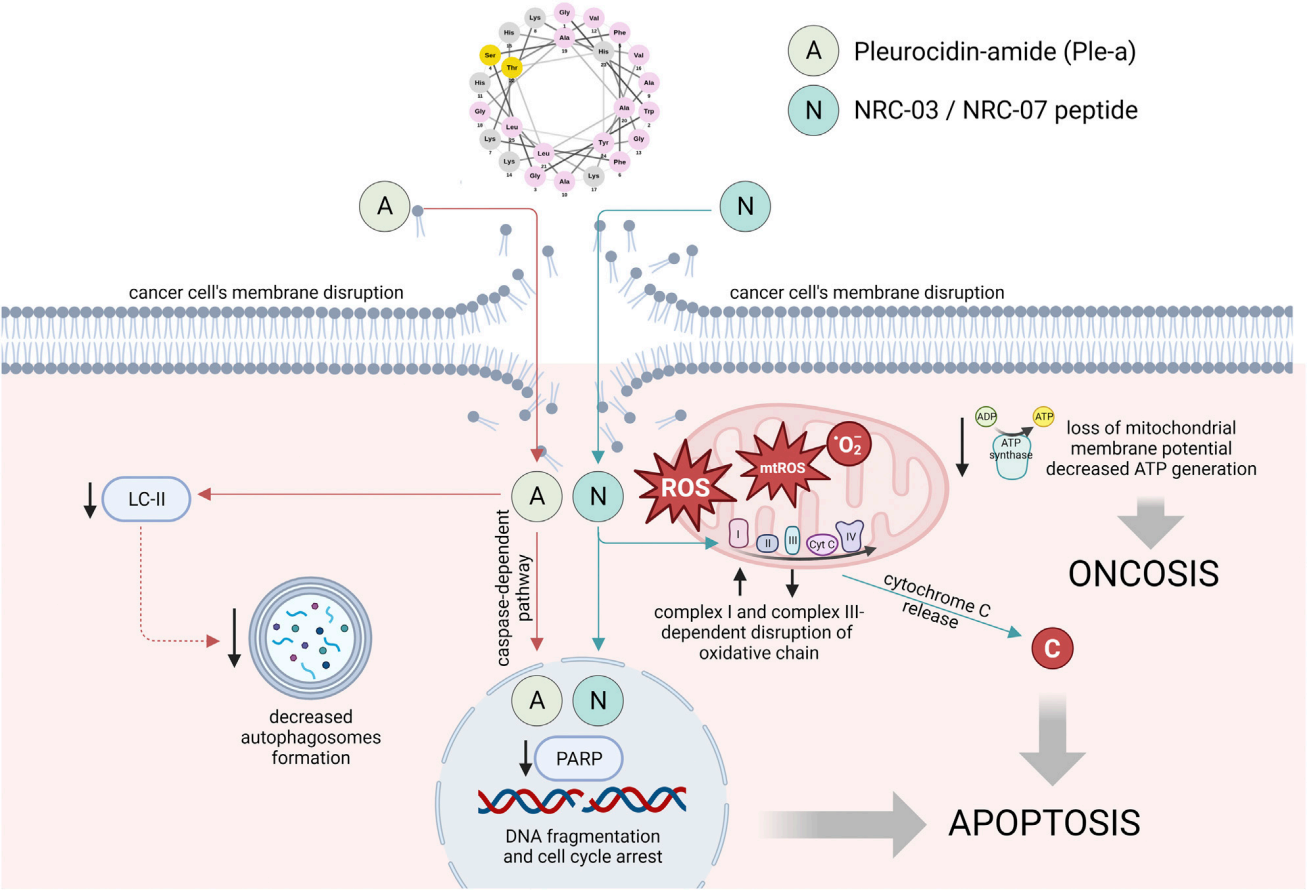
The main mechanisms of anti-cancer activities of pleurocidin-amide (Ple-a) and NRC-03/NRC-07 peptides. The mode of action of Ple-a involves the induction of apoptosis, followed by the suppression of autophagy during the late stage of cell death. The localization of NRC-03 and NRC-07 peptides in both nuclei and mitochondria was observed, accompanied by their cytotoxic effects through both cytoplasmic and mitochondrial ROS-dependent mechanisms. These mechanisms led to the impairment of intracellular structures and reduced intracellular energy reserves, ultimately resulting in apoptosis and induction. Figure prepared using Biorender.com.

To date, the available data on the anti-cancer properties of native pleurocidin is limited. In most previous studies, Ple was used as a starting agent for other synthetic analogs and Ple-like peptides with potent anti-cancer activities. Only a single study has provided evidence supporting the diverse characteristics of pleurocidin-amide, including its potential application as an anti-cancer therapeutic agent against hepatocellular carcinoma as well as non-small cell lung adenocarcinoma, stomach adenocarcinoma, and colon adenocarcinoma (Hsu et al., 2022). An amidated derivative of Ple when tested against different cell lines was characterized by several-fold lower values of IC_50_ (ranging from 11 to 197.3 µM) when compared to the control peptide (from 54.9 to >500 µM) (Hsu et al., 2022). evidenced, Ple-a affected the cell cycle by increasing the number of cells in the sub-G1 phase and induced apoptosis, as evidenced by a decrease of pro-form PARP. At a late stage of treatment, Ple-a-mediated apoptosis was additionally enhanced by inhibition of autophagy (Hsu et al., 2022) (Figure 2).

Subsequently, in contrast to a limited amount of data on the anti-cancer activities of pleurocidin, a substantial body of evidence has been generated regarding the anti-malignant capabilities of Ple-like peptides. In 2003, Patrzykat et al., 2003 conducted a study in which they employed a genetic methodology to identify a set of pleurocidin-like peptides, which had similar signal and pro-region sequences while displaying variations in their core antimicrobial sequences and thus, varied biological activities. Based on this, a total of 20 peptides were produced synthetically and denoted as NRC-01 to NRC-20. Peptides underwent screening to assess their antimicrobial characteristics, but subsequent investigations afterward validated their considerable cytotoxic capacity (Hilchie et al., 2011). Importantly, among all produced peptides, only 4 were demonstrated to be virtually inactive and others were classified as those with a broad-spectrum or more limited spectrum of activity (Patrzykat et al., 2003). In the following investigations, Morash et al., 2011 increased the pool of inactive peptides to 14 (from the 26 NRC peptides that were screened), however, it might be agreed upon that further investigation is necessary to thoroughly evaluate the potential of these peptides. NRC-03, NRC-12, and NRC-13 were recognized as the most active, and indeed, a majority of studies on the anti-cancer activities of these compounds are focused on the NRC-03 peptide (Hilchie et al., 2013; Hou et al., 2022) (Figure 2).

In one of the first studies, a total of 26 NRC peptides (designated as 1–20 and 123–128) were screened alongside an enantiomer of NRC-03 against human leukemia HL60 cells and human erythrocytes to explore the anti-cancer potential of this group of compounds (Morash et al., 2011). Peptides that were classified as the most active (lytic activities no higher than 32 μg/mL) exhibited a characteristic amphipathic α-helical conformation and were positively charged (at least +6.5). NRC-03 was demonstrated to display cytotoxic effects against cancer cells *via* the release of excessive ROS from impaired mitochondria followed by decreased intracellular energy reserves, leading ultimately to damage of DNA and membrane permeabilization, thus acting by oncosis and apoptosis mechanisms. Particularly, induction of oncosis, which is a process manifesting within a brief timeframe of seconds to minutes subsequent to the cellular injury or plasma membrane impairment (Trump et al., 1997) gave hope for the low rate of resistance of cancer cells to these peptides, although other reports suggest some caution for such conclusions (Hilchie et al., 2020). In another study, NRC-03 and NRC-07 were presented to selectively kill breast carcinoma cells both *in vitro* and *in vivo.* Importantly, NRC peptides were effective against cancer cells that exhibited drug resistance due to overexpression of P-glycoprotein, as well as against slow-growing tumor cells suggesting the potential therapeutic value against indolent tumors. Suggestions were also made that NRC-03 and NRC-07 display dual mechanisms of cell death upon prolonged exposure to lower concentrations of agents, as was presented previously on bacteria (Patrzykat et al., 2002), although this was not confirmed experimentally. Notably, sublethal concentrations of NRC-03 improved also the effectiveness of cisplatin, proposing the potential utilization of the peptide as a chemosensitizing agent by a mechanism involving facilitated uptake of cisplatin (Hilchie et al., 2011). In agreement with these reports, NRC-03 and NRC-07 peptides were demonstrated to rapidly kill myeloma cells by causing extensive membrane damage, as well as DNA cleavage. Furthermore, intratumoral injections of NRC-03 impaired the growth of multiple myeloma xenografts in immune-deficient mice. In contrast to the breast cancer cells, which exhibited similar sensitivity to both tested NRC peptides, the multiple myeloma cells demonstrated a higher susceptibility to cytotoxic effects induced by NRC-03. This observation is consistent with the significantly stronger binding affinity of NRC-03 towards multiple myeloma cells, which was found to be 3–4 times greater (Hilchie et al., 2013). Most recently, NRC-03 peptide was also demonstrated to kill oral squamous cell carcinoma cells by a mechanism involving increased oxygen consumption rate followed by overexpression of genes encoding complex I subunits with subsequent decrease of complex III subunit’s genes expression, loss of mitochondrial membrane potential, and CypD-mPTP axis-mediated decrease of ATP production leading ultimately to apoptosis induction (Hou et al., 2022). In effect, the statements on the multifaceted activities of this peptide against cancer cells and its great applicability in the treatment of malignancies were encouraged.

Regrettably, the therapeutic efficacy of NRC peptides may be compromised due to their vulnerability to proteolytic degradation. Specifically, NRC-03 is susceptible to breakdown when exposed to trypsin and its cytotoxicity is lowered in the presence of serum (Hilchie et al., 2011). For this purpose, efforts are made to develop the active variants of pleurocidin-derived peptides, including NRC ones, which are resistant to proteases and thus applicable for the treatment of infections and malignancies (Jung et al., 2007; Morash et al., 2011; Hilchie et al., 2015). Such an approach includes development mostly the developing of peptides consisting of D-amino acids analogues and the results of these findings are contradictory. Specifically, the NRC-03D peptide, which incorporates D-lysine and D-arginine amino acids to mitigate protease digestion and improve stability was reported to lose its anti-cancer activity against human leukemia HL60 cells (IC_50_ > 128 μg/mL) (Morash et al., 2011). In contrast to that, [D]-NRC-03 peptide in which all of the L-amino acid residues were replaced with D-amino acids displayed enhanced potency against various breast cancer cell lines regardless of serum concentration (Hilchie et al., 2015). Interestingly, such amino acid substitutions affected also the kinetic of killing action (for [D]-NRC-03 delayed kinetic was noted). Collectively, the development of variants with enhanced stability in protease environment with subsequently increased selectivity for cancer cells is therefore required and open for further research.

One intriguing strategy for utilizing pleurocidin-derived peptides involves the development of chimeric proteins composed of two peptides, potentially leading to synergistic cytotoxic effects. The first such attempts were made by Soleimani *et al.* and were focused on the creation of dual-agent protein consisting of p28 and NRC peptides, both characterized by cytotoxic activities with varied mechanisms of action, thus potentially displaying enhanced anti-cancer effects then applied together (Soleimani et al., 2016a; Soleimani et al., 2016b). The applicability of such developed chimeric protein was presented in the later study using an *in vitro* model of breast cancer. p28-NRC exerted its anticancer effects on MCF7 breast cancer cells through mitochondrial caspase-dependent and -independent apoptotic pathways and the observed effects were significantly more potent than those observed for single peptides, providing a rationale for further investigation in this area of research (Soleimani et al., 2019). Combining of NRC-03 peptide with other biologically active molecules was also engaged to develop a multi-compound nanocomposite for enhanced efficient near-infrared (NIR) photothermal therapy of breast cancer (Chen et al., 2019). By conjugation of NRC-03 to polydopamine (pDA)-modified reduced graphene oxide (rGO) a nanoformulation with excellent NIR optical absorbance, improved stability, high biocompatibility, and improved anti-cancer efficiency (determined by a burst release of the NRC-03 peptide from NRC-03-pDA/rGO upon photothermal conditions) was obtained opening the discussion of novel NRC-containing conjugates for augmented therapy of breast cancer (Chen et al., 2019).

Although NRC peptides are the most recognized and well-studied group of pleurocidin-derived compounds with anti-cancer potential, other peptides were also successfully tested against most clinically-relevant malignancies. Most recently, pleurocidin-like peptide WF3 (AMP-WF3) isoform X2 isolated from *Poecilia mexicana* fish was presented to exhibit cytotoxicity and anti-proliferation capabilities toward acute lymphoblastic Jurkat cell line by mechanisms involving activation of *p21* and *p53-*mediated signaling pathways, cell cycle arrest and induction of cell apoptosis (Ebrahimdoost et al., 2023). The aforementioned observation provides compelling evidence that the pleurocidin-like peptide possesses the ability to exert various effects on cellular viability and selectively target a range of intracellular components, which presents novel avenues for further investigation into the cell selectivity of Ple and its derived analogs.

## 6 Cancer cells’ selectivity of pleurocidin-derived peptides

Thus far, there is a substantial body of research demonstrating the cell selectivity of pleurocidin and pleurocidin-derived peptides. Originally, cell selectivity of Ple and its analogs was demonstrated for mammalian versus bacterial cells, however, there is a growing body of evidence suggesting the selectivity of these Ple-like compounds towards cancer cells over non-malignant cells, and thus, supporting the notion of potential utility of these compounds in cancer treatment. As such, Hsu et al., 2022 demonstrated that both Ple and Ple-a were considerably less cytotoxic against mouse fibroblasts NIH-3T3 cell line than against liver, lung, stomach, and colon cancer cells. This phenomenon was particularly prominent for hepatocellular carcinoma J5 cells, which were noted to be nearly 30-fold more prone to applied treatment than non-malignant cells. In another study aiming to screen NRC peptides for activity against leukemia cells, strong evidence for cancer cell selectivity of NRC-03 was provided, as human mammalian epithelial cells (HMECs) even upon prolonged exposition to NRC-03 did not display considerably intensified effects of toxicity, in contrast to HL60 cells for which alterations in morphology and membrane integrity were noted shortly upon peptide addition. Moreover, IC_50_ of NRC-03 was 4-fold higher against human vascular endothelial cells (HuVEC) than against HL60, and hemolysis was not detected at the highest concentration tested (256 μg/mL) (Morash et al., 2011). NRC-03 and NRC-07 were demonstrated also by Hilchie et al., 2011 to be non-toxic against non-target human erythrocytes and did not exhibit cytotoxic effects on primary cultures of human dermal fibroblasts or HUVECs at concentrations that were highly cytotoxic for breast cancer cells. Such effect was concluded as resulting from a substantially greater binding capacity of peptides to breast cancer cells than to normal fibroblasts. Nonetheless, primary cultures of human mammalian epithelial cells (HMECs) exhibited significant cytotoxicity when exposed to both NRC-03 and NRC-07, although the observed cytotoxicity was comparatively lower than that observed in breast cancer cells. At the same time, a satisfactory safety profile was established by Ebrahimdoost et al., 2023 for pleurocidin-like peptide WF3 isoform X2 (AMP-WF3) toward tested non-cancerous cells, i.e., peripheral blood mononuclear cells (PBMCs) and human dermal fibroblast (HDF) cells. Although such phenomenon might be directly linked with a different composition of Jurkat cells (containing high levels of cholesterol, phosphatidylserine and other anionic components on the outer lipid membrane) and normal cells (characterized by high levels of phosphatidylcholine and sphingomyelin, which has zwitterionic properties), it is important to note that the activation patterns of the *p21*, *p53,* and *Bcl-2*-mediated pathways differed significantly between cancerous and normal cells. At the same time, caution should be taken when modifying the structure of Ple-like peptides to obtain more proper pharmacokinetic or pharmacodynamic features. As such, the replacement of L-amino acids with D-amino residues might affect the toxicity against non-cancer cells, as evidenced by Hilchie et al., 2015. Accordingly, when comparing [L]- and [D]-NRC-03 peptides’ cell selectivity it was demonstrated that although the low level of hemolysis was maintained for both enantiomers, [D]-NRC-03 peptide was recorded to be more toxic against blood mononuclear cells, HMECs, human dermal fibroblasts, and HUVEC cells than its parent peptide, although reasons of such lower selectivity were not clear (Hilchie et al., 2015). Notably, this was demonstrated only *in vitro* and there were no apparent indications of its occurrence recorded in the mouse model (Hilchie et al., 2015). Collectively, pleurocidin-like peptides offer great potential to be used as novel anti-cancer therapeutics as they are characterized by limited toxicity against non-transformed cells both in artificial cell culture-based experimental settings and animal models.

## 7 Induction of cancer cell drug resistance against Ple-like peptides

The characteristics of endogenous anti-cancer peptides suggest that the likelihood of cancer cells developing drug resistance to therapeutics based on these peptides is significantly impeded and less likely compared to conventional cytotoxic agents (Deslouches and Di, 2017; Jafari et al., 2022). The topic concerning the development of drug resistance in bacterial and cancer cells when exposed to pleurocidin and pleurocidin-derived peptides remains an area that requires further investigation, although certain findings may indicate potential outcomes following extended exposure to these peptides. The initial investigation of pleurocidin antimicrobial properties revealed that *Leucothrix mucor*, Gram-positive fish-host bacteria indigenous to the surface of winter flounder eggs exhibited resistance to Ple-mediated killing (MIC/MBC values >35 µM), which suggests a bacterial mechanism that has evolved to counteract Ple antimicrobial attack (Cole et al., 1997). In another study, *Lactobacillus acidophilus* was also demonstrated to be even 16 to 64-fold less susceptible to pleurocidin action compared to other oral microorganisms (Tao et al., 2011). Species of bacteria that are particularly resistant to Ple include *Enterococcus faecalis*, a commensal microbe of the mammalian gastrointestinal tract although the reports on this issue might be contradictory depending on the peptide derivative and bacterial strain tested (Tao et al., 2011; Choi and Lee, 2012; Souza et al., 2013). These reports indicate that the occurrence of decreased susceptibility to Ple is possible and should be taken into consideration although the source of such phenomenon was not explored to date. When related to cancer cells, only one research aiming to investigate this topic was performed using NRC-03 and NRC-07 peptides as therapeutic agents against breast cancer cells (Hilchie et al., 2020). In contrast to prevailing viewpoints regarding the inability of cancer cells to develop drug resistance against cytolytic anti-cancer peptides, Hilchie *et al.* generated two variants of MDA-MB-231 breast cancer cells with diminished susceptibility to the applied treatment and demonstrated a correlation between induced peptide resistance and a reduction in peptide binding to the cell membrane, suggesting that resistance is attributed to changes in the composition of the cell membrane. The confirmation of changes in the expression of genes related to angiogenesis, interactions with the extracellular matrix (ECM), and antigen processing and presentation was also established. Significantly, breast cancer cells that were resistant to NRC exhibited different phenotypic characteristics, while still maintaining susceptibility to chemotherapeutic treatments. It is worth mentioning that both breast cancer cell lines resistant to peptides exhibited an inability to form tumors in immune response-lacking mice, which indicates that the modifications required to decrease vulnerability to peptide-induced cytotoxicity also significantly impede tumor development (Hilchie et al., 2020). Based on this, the occurrence of the development of resistance of target cells to pleurocidin and pleurocidin-derived peptides is plausible, nevertheless, due to the limited data on this subject, further research is necessary to comprehensively grasp the implications of this phenomenon.

## 8 What are potential avenues for future research in the exploration of pleurocidin-derived anticancer therapeutics?

Although some compelling data on the potential anti-cancer activities of pleurocidin and pleurocidin-derived peptides are available (Hilchie et al., 2013; Soleimani et al., 2019), there are also several limitations and drawbacks of this group of compounds that could be optimized and improved to develop fully successful anti-cancer therapeutics. One area of focus involves the refinement of the molecular structure of pleurocidin-derived compounds to further improve their anti-cancer properties and increase their selectivity towards cancer cells. Such an approach has been employed on multiple occasions to enhance the stability and antibacterial efficacy of Ple-like compounds. However, it is worth noting that only a limited number of studies have specifically investigated the identification of the most cytotoxic fragments of Ple or explored how modifying the amino acid sequence could enhance antineoplastic effects. Moreover, the in-depth investigation of the molecular effects of pleurocidin and pleurocidin-derived peptides, as well as the identification of intracellular targets and putative cellular receptors, is of paramount importance as these data will help to understand the activity and dependency of these molecules. For this purpose, *in silico* investigations would be helpful and have the potential to elucidate strategies for altering endogenous peptides to produce targeted cytotoxic effects.

Another issue requiring resolution is the susceptibility of Ple and Ple-like peptides, which are mostly composed of L-amino acids, to proteolytic enzymes which might considerably affect their effectiveness upon systemic administration (Hilchie et al., 2015). In light of this rationale, endeavors have been undertaken to synthesize enantiomers of pleurocidin and NRC peptides to achieve heightened stability, hence augmenting their antibacterial and anti-cancer efficacy (Jung et al., 2007; Morash et al., 2011; Hilchie et al., 2015; Wang et al., 2022). Nevertheless, as evidenced, the outcomes of implementing this method are not consistently advantageous (Morash et al., 2011; Hilchie et al., 2015). The nonspecific toxicity of certain D-enantiomers can be substantially reduced by the addition of cancer cell-targeting moieties (Leuschner et al., 2003; Hansel et al., 2007) or the substitution of arginine and lysine residues with histidine residues allowing pH-dependent activation in the acidic tumor microenvironment since histidine develop a positive charge under the acidic conditions (Makovitzki et al., 2009). Nonetheless, the latter approach also has to be extensively tested as the protonation of histidine residues has been shown to have no impact on the membrane-disrupting effect of pleurocidin or the peptide’s positioning within the membrane, as evidenced under acidic pH conditions (Mason et al., 2006). Alternatively, the cancer cell selectivity of developed enantiomers might be also improved by binding them with the moieties that target cancer cell-overexpressed molecules (Akhtar et al., 2014). They might be linked directly to the anti-cancer peptides or conjugated together with other biologically active components on the surface of nanocarriers (Piktel et al., 2016) but in such cases, a size-dependent decrease in tumor penetration and alterations in pharmacokinetics and biodistribution or increase of toxicity should be taken under the consideration (Kuna et al., 2018). Moreover, a plethora of chemical modifications to provide protease shielding, including backbone modification, cyclization or incorporation of amino acids with non-canonical side chains should be recognized (Lucana et al., 2021), although, for pleurocidin and Ple-derived peptides, a majority of them have not tested to date. It opens new possibilities to optimize novel peptide-based therapeutics, however, such effects as altered homing and transport efficiency should also be comprehensively explored (Lucana et al., 2021).

Furthermore, it is important to give careful attention to the assessment of whether pleurocidin can induce anti-cancer effects through indirect modes of action. A plethora of host defense peptides with anti-cancer activities was proven to limit tumor growth and metastasis *via* mechanisms other than direct cytotoxicity (Tripathi and Vishwanatha, 2022; Chinnadurai et al., 2023). As such, D-K6L9 peptide, an engineered membranolytic anticancer peptide made of lysine and leucine amino acids, was demonstrated to reduce neovascularization upon administration to immunodeficient, tumor-bearing mice (Cichoń et al., 2014). Lactoferrin was also reported to suppress tumor angiogenesis *via* inhibition of NF-κB signaling pathways (Ayuningtyas et al., 2023). In the context of pleurocidin and pleurocidin-derived peptides, to date, no convincing data on such mechanisms were noted and only Hilchie et al., 2020 *.* demonstrated NRC-03/NRC-07-mediated alteration in angiogenesis-involved genes. Regretfully, the correlation between NRC-03 and NRC-07-induced cytotoxicity and angiogenesis remains elusive (Hilchie et al., 2020).

Unexplored areas are also the anti-inflammatory properties of pleurocidin and pleurocidin-derived peptides. The crosslink between inflammation and cancer progression is well-recognized and widely accepted (Greten and Grivennikov, 2019; Singh et al., 2019). Consequently, there is an increasing recognition within clinical settings regarding the therapeutic capabilities of interventions that can modulate immune responses and attenuate inflammation thus, serving as chemoprotectants or as chemosensitizers to conventional cancer therapies (Rayburn et al., 2009). To date, data are scarce about the influence of Ple and Ple-derived peptides on immune response and inflammatory profiles, nevertheless, it is possible to speculate about potential implications for anti-cancer treatments. In one of the studies, Pundir et al., 2014 demonstrated that out of 20 peptides of the NRC class (from NRC-01 to NRC-20), 11 peptides exhibited the ability to induce mast cell degranulation and NRC-04 revealed the highest level of potency in this regard. More detailed analyses demonstrated the NRC-04-mediated release of preformed granule-contained mediators and stimulation of the production of chemokines *via* N-formyl-peptide receptor 1 (FPRL1) receptor signaling. While this phenomenon was revealed only for human mast cells (Pundir et al., 2014), a question is left how Ple-like compounds affect the functions of other immunocompromised cells, including NK cells. In a later report, the utilization of D-leukocidin-KR, which involved the substitution of lysine residues with arginine, was proposed as a means to impede pro-inflammatory reactions in a mouse model of lung infection. Nevertheless, it was understood that these benefits were contingent upon the direct bactericidal properties exhibited by this peptide (Manzo et al., 2020). Likewise, pleurocidin was demonstrated to induce the expression of inflammatory genes, i.e., *IL-1*β and COX-2 in the trout macrophage RTS11 cells, but it was not able to adversely affect the LPS-induced effects on the expression of these genes (Peter Chiou et al., 2006). To date, there has been a lack of exploration into the impact of pleurocidin and NRC-group peptides on the production of cytokines and chemokines in the context of cancer. This existing knowledge gap presents a promising avenue for future research endeavors in this particular domain.

## 9 Conclusion

Presently, there is a growing interest in developing pleurocidin and pleurocidin-derived peptides as anti-cancer therapeutics. For such purposes, microbiological studies exploring the structural and physicochemical characteristics of pleurocidin are particularly useful. To date, some promising data on cytotoxic activities of Ple and Ple-derived peptides, particularly those from the NRC peptides group, were demonstrated, giving new hope for the identification of potent and cancer cell-selective molecules. However, further research is required to comprehensively understand the clinical importance of pleurocidin and pleurocidin-derived peptides. This includes additional studies on the non-membranolytic mechanisms of action of pleurocidin, as well as investigating their toxicity profile and potential for developing drug resistance.
